# Intertoe Squamous Cell Carcinoma Developed in a Patient with Rheumatoid Arthritis under Etanercept Therapy

**DOI:** 10.1155/2015/315490

**Published:** 2015-03-01

**Authors:** Efstathios Rallis, Vasiliki-Kalliopi Bournia, Constantinos Verros, Alexios Iliopoulos

**Affiliations:** ^1^Department of Dermatology, Veterans Administration Hospital (NIMTS), Monis Petraki 10-12, 11521 Athens, Greece; ^2^Department of Rheumatology, Veterans Administration Hospital (NIMTS), Monis Petraki 10-12, 11521 Athens, Greece; ^3^Private Practice, Tripolis, Greece

## Abstract

The use of tumor necrosis factor-*α* (TNF-*α*) inhibitors in the treatment of various inflammatory conditions has altered the field of medical therapeutics. Squamous cell carcinoma is the second most common cancer of the skin, usually affecting sun-exposed areas of the body. We present here the case of a 75-year-old woman with rheumatoid arthritis, who developed an intertoe squamous cell carcinoma (SCC) of the right foot. According to her history, she received etanercept and methotrexate for 5 years for rheumatoid arthritis. The rare localization of this cancer could suggest a possible linkage of the malignancy to the chronic intake of anti-TNF-*α* treatment. This is the first reported case of an interdigital SCC developed under the use of an anti-TNF-*α* agent.

## 1. Introduction

Tumor necrosis factor-*α* (TNF-*α*) inhibitors have revolutionized the treatment of inflammatory diseases during the last decade. Nevertheless, concerns have been expressed in literature, regarding a possible carcinogenetic potential of these drugs, particularly with respect to non melanoma skin cancers [1, 2].

Squamous cell carcinoma (SCC) is the second most common cancer of the skin in Caucasian populations, following basal cell carcinoma. It rises from the keratinocytes of the epidermis and has a predilection for sun-exposed areas of the body, primarily the head and neck [3].

We present the case of a rheumatoid arthritis (RA) patient, who developed an intertoe SCC under long treatment with etanercept and methotrexate. The rare localization of this cancer could suggest a possible linkage of the malignancy to the immunomodulatory effect of this treatment.

## 2. Case Report

**Figure 1:**
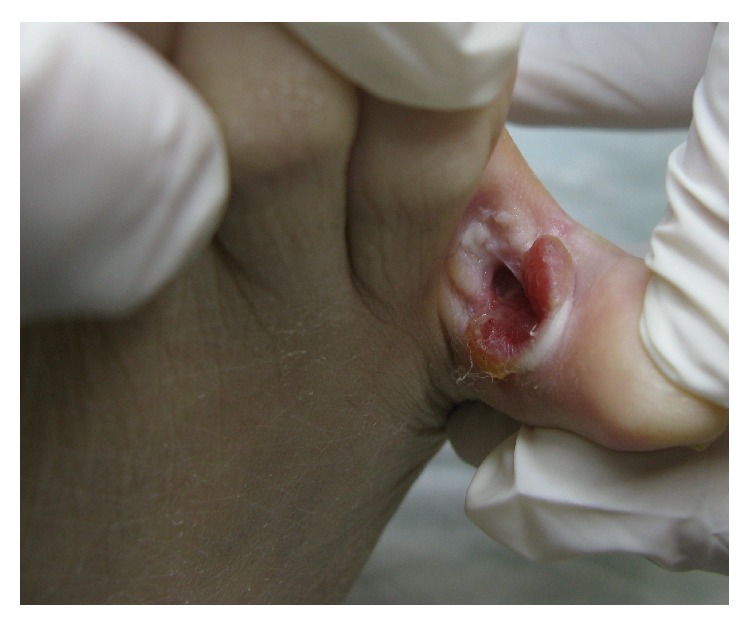
A deep ulcer located on the fourth intertoe space of the right foot.

A 75-year-old woman, non smoker, with a known history of RA, presented to the dermatology department of our hospital with a persistent ulcer on the fourth intertoe space of the right foot (Figure 1). The patient was receiving etanercept 50 mg per week, prednisolone, and methotrexate for the past five years and her arthritis was currently in remission. According to her history, she firstly noticed a small ulcer on her foot, approximately ten months ago. She consulted her physician, who considered it as a local infection and prescribed local antimicrobial and antifungal treatment, with no improvement.

**Figure 2:**
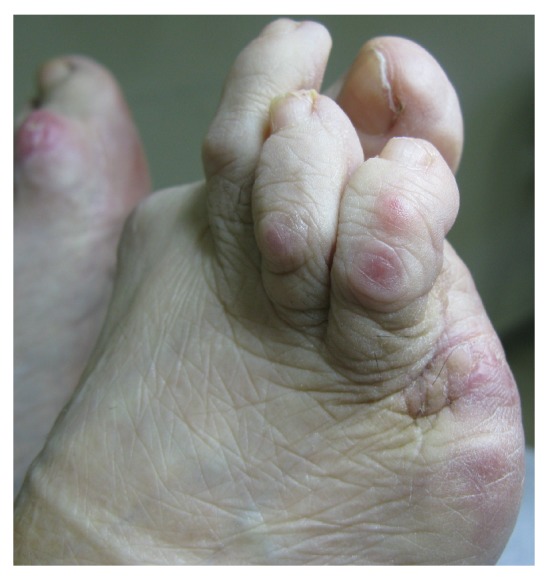
Postoperative photo of the right foot. The lesion has been excised on healthy boundaries with removal of the fifth toe.

During the next months the ulcer slowly progressed and she was referred to our hospital, where she underwent mycologic and bacteriologic examination. Gram stains and cultures obtained from the ulcer were negative. A 3 mm punch biopsy of the lesion was performed and histological examination revealed the presence of a SCC. The lesion was surgically excised on healthy boundaries with removal of the fifth toe (Figure 2). The patient's further course was uneventful.

## 3. Discussion

The relationship between TNF-*α* inhibitors and the risk for malignancy is hard to establish, given that most patients on this treatment are inherently predisposed to certain forms of cancer due to their underlying disease [4]. In a meta-analysis of 33 double-blind, randomized placebo controlled trials of infliximab, etanercept, adalimumab, golimumab, and certolizumab in adult patients with RA, no excess risk for malignancy was revealed, either in the per protocol or in the intention to treat analysis. However, the authors reported a nonsignificant tendency for an excess non melanoma skin cancer risk in both models [5].

Recently, an update on the evidence for the safety of synthetic disease modifying antirheumatic drugs (sDMARDs), glucocorticoids, and biological DMARDs (bDMARDs) in patients with RA was reported [6]. This systematic review of the literature evaluated 49 observational studies. Patients on TNF-*α* inhibitors compared to patients on conventional sDMARDs did not have a higher risk for malignancies in general, lymphoma or non melanoma skin cancer, but the risk of melanoma could be slightly increased.

Several case reports have previously associated anti-TNF-*α* treatment with the development of SCCs, which were often multiple and of rapid onset. These carcinomas were mostly located on the lips, face, neck, chest, upper back, arms, genitalia, and pretibial areas [7–11], but to the best of our knowledge, there is no report of an interdigital SCC.

Intertoe space SCCs, unrelated to immunosuppressive or anti-TNF-*α* treatment and to HPV infection, have been previously described in only two publications. They were both from France: a case report of a 66-year-old woman [12] and a case series of 22 intertoe SCCs in 19 patients [13]. These two articles considered continual maceration as the key aetiological feature for the lesion.

In our patient, the age was probably the only predisposing risk factor for the development of SCC [3]. The rare localization of the tumor on a non-sun-exposed area and the well-established correlation of SCCs to immunosuppression [3] could possibly imply a link of the malignancy to the lengthy use of anti-TNF-*α* and methotrexate for her underlying RA. Additionally, the rare localization delayed the prompt diagnosis, allowing the progression of the tumor and leading to the removal of her fifth toe.

Based on the experience, we stress the need for vigilance of the physicians for refractory ulcers especially in patients receiving immunomodulatory treatments including TNF-*α* inhibitors.
